# Protein Detection with Potentiometric Aptasensors: A Comparative Study between Polyaniline and Single-Walled Carbon Nanotubes Transducers

**DOI:** 10.1155/2013/282756

**Published:** 2013-02-26

**Authors:** Ali Düzgün, Hassan Imran, Kalle Levon, F. Xavier Rius

**Affiliations:** ^1^Department of Analytical and Organic Chemistry, Universitat Rovira i Virgili, Campus Sescelades, Marcel*·*lí Domingo, 43007 Tarragona, Spain; ^2^Department of Chemical and Biological Sciences, Polytechnic Institute of New York University, Brooklyn, NY 11201, USA

## Abstract

A comparison study on the performance characteristics and surface characterization of two different solid-contact selective potentiometric thrombin aptasensors, one exploiting a network of single-walled carbon nanotubes (SWCNTs) and the other the polyaniline (PANI), both acting as a transducing element, is described in this work. The molecular properties of both SWCNT and PANI surfaces have been modified by covalently linking thrombin binding aptamers as biorecognition elements. The two aptasensors are compared and characterized through potentiometry and electrochemical impedance spectroscopy (EIS) based on the voltammetric response of multiply charged transition metal cations (such as hexaammineruthenium, [Ru(NH_3_)_6_]^3+^) bound electrostatically to the DNA probes. The surface densities of aptamers were accurately determined by the integration of the peak for the reduction of [Ru(NH_3_)_6_]^3+^ to [Ru(NH_3_)_6_]^2+^. The differences and the similarities, as well as the transduction mechanism, are also discussed. The sensitivity is calculated as 2.97 mV/decade and 8.03 mV/decade for the PANI and SWCNTs aptasensors, respectively. These results are in accordance with the higher surface density of the aptamers in the SWCNT potentiometric sensor.

## 1. Introduction

Biosensors based on electrochemical detection have been extensively used to detect proteins [1–3]. They offer, in addition to selectivity and sensitivity, the possibility to detect the target analytes in cloudy samples in a very simple and fast way. Even though the electrochemical techniques employed, such as amperometry, voltammetry, or electrochemical impedance spectroscopy (EIS), provide these performance characteristics, the relative complexity of the detection procedures and the need for portable detectors enabling the detection of the targets at the point of care motivate the development of more rapid, cheaper, and simpler detection techniques. 

Potentiometry is one of the most simple electrochemical detection methods. Nanostructured biosensors based on field effect transistors (FETs) are considered members of this type [4, 5]. The miniaturized bio-FETs are able to detect nowadays large molecules such as plasma proteins or even bacteria [6, 7]. However, these devices display low physical robustness, large response times, and poor reproducibility among individual sensors. Moreover, they are usually developed using microfabrication techniques, and consequently they display high production costs. The appearance of potentiometric all-solid-state aptasensors other than FETs made it possible to overcome most of these problems [8].

Aptamers enable the development of cheap and sensitive biosensors. Aptasensors, thanks to their relatively reduced nucleic-acid-based nature, display several advantages over the antibody counterparts developed for the same targets: higher heat, pH and ionic strength stability, smaller size, and in some cases higher selectivity [9]. Moreover, they can be synthesized at low cost. Electrochemical biosensors incorporating aptamers as recognition elements are extensively reported in the bibliography [10–14], although the translation to commercialized devices is very scarce [15].

Düzgün et al. recently demonstrated the feasibility to potentiometrically detect large analytes such as proteins using a nanostructured hybrid material (based on carbon nanotubes, CNTs) that incorporates thrombin binding aptamers (TBAs) [16]. The main advantages of this detection system are simplicity due to the two-electrode system used in potentiometry, low cost, and real-time detection which make it highly valuable for different types of applications. Zelada-Guillén et al. showed that the same strategy could be applied to quantify bacteria in real samples [17, 18]. The biosensing mechanism is thought to be based on the superficial restructuration of the aptamers lying onto the surface of the single-walled carbon nanotubes (SWCNTs) when the target analyte, displaying a very high affinity constant with the aptamers, enters in contact with them. Johnson et al. [19] have recently demonstrated that aptamers are self-assembled to carbon nanotubes via **π*-*π** stacking interaction between the aptamer bases and the carbon nanotubes walls by using molecular dynamics. Since the phosphate groups of the aptamers are largely ionized at pH 7.5, these negative charges can be transferred to the carbon nanotubes. This agrees with the decrease in the initial potential of the sensor measured following the functionalization of the SWCNTs with the aptamers. The presence of the target protein induces a conformational change in the aptamer that separates the phosphate negative charges from the SWCNT side walls [20] inducing the subsequent increase of the recorded potential. This mechanism is similar to the one reported by Levon's group in the development of a nucleic acid potentiometric biosensor based on the hybridization of the complementary DNA strands and using polyaniline (PANI) as a transducer layer [21]. The sensing mechanism was assigned to a different interaction of the nucleic acid probes with the strongly cationic polyaniline substrate. The conformational changes in the nucleic acid probes caused by the selective hybridization with the complementary strand provide the potential change that is monitored. 

Due to the similarity of the proposed sensing mechanisms, it would be interesting to compare between the performances of the conducting polymers and the SWCNTs as transducer elements in these potentiometric sensors. PANI and SWCNTs show different characteristics in terms of material nature, electrical conductivity, deposition procedures, and thickness control of the transducing layer. Therefore, it is worthwhile that the comparison of their performance characteristics could provide us with an advantage in terms of producing similar performance characteristics considering the relatively simple spraying method that is used for SWCNTs. 

The characterization of the aptamer-SWCNT-based aptasensors, basically the number of aptamers linked for unit length of carbon nanotube, is difficult due to the specific nature of the substrate and the small size of the nucleic acid segments attached to the carbon nanotube walls. Electrochemical techniques could provide a suitable methodology, although EIS studies cannot be applied directly to the system due to the reduced conductivity on the sensor surface made of semiconducting SWCNTs [22]. Surface ligand density calculation as a part of electrochemical characterization is a key factor in determining the source of the potentiometric signal, as the classical Nernstian theory is not applicable due to the lack of a thermodynamic equilibrium at the sensor surface.

In this work, we conducted a comparative study of the aptasensors to determine protein using both SWCNT and PANI. We compared the sensitivity and the stability of the sensors using TBA as a recognition layer and human alpha thrombin as a target analyte. Furthermore, we characterized the solid surface by measuring the total surface aptamer density based on the Cottrell equation [23, 24] assuming complete charge compensation of the DNA phosphate residues by redox cations. 

## 2. Material and Methods

### 2.1. Instrumentation and Reagents

A Lawson (USA) multichannel potentiometer and a Metrohm (Switzerland) Ag/AgCl reference electrode were used to perform the potentiometric experiments. A Metrohm (Switzerland) laboratory type pH probe was used for the pH detection using 3-standard calibration. A CHI Instruments (USA) electrochemical workstation was used for cyclic voltammetry (CV) experiments. An FEI Company (Netherlands) SEM-Quanta 600 is used to take the SEM image. Aniline monomer, [Ru(NH_3_)_6_]^3+^ (hexaammineruthenium, Ru[(NH_3_)_6_]Cl_3_), and H_2_SO_4_ were purchased from Sigma-Aldrich (Spain). Single-walled carbon nanotubes (SWCNTs) were purchased from HeJi (China) in bulk form with >90% purity, 150 *μ*m average length, and 1.4–1.5 nm of diameter. 15-mer (5′-GGTTGGTGTGGTTGG-3′) 5′-NH_2_ and 15-mer (5′-GGTTGGTGTGGTTGG-3′) 5′-SH modified (with a 3-carbon spacer) thrombin binding aptamers (TBAs) were purchased from Eurogentec (Cultek, Spain) and Genemed Synthesis Inc. (USA), respectively. Human *α*-thrombin was supplied by Haematologic Technologies (Vermont, USA). Elastase and BSA were supplied by Aldrich. Phosphate buffer solution (PBS) is purchased from Panreac Química (Spain). Glassy carbon (GC) rods were purchased from HTW (Germany). Aniline monomer (Aldrich) was distilled and kept cooled at 4°C. It was also kept in the dark to avoid any potential photo-oxidation. Sodium dodecyl sulphate (SDS) (Aldrich) was used as purchased.

### 2.2. Sensor Preparation

The solid contact sensors were prepared by placing a 3 mm diameter glassy carbon rod into a teflon body with the outer diameter of 7 mm. The tip was polished firstly using a Buehler p4000 paper. Subsequently, 6 *μ*m diamond polish and 1 *μ*m grain size alumina powder were used to obtain a smooth surface. Polished sensors were bath sonicated for 30 minutes in Milli-Q water to clean the alumina and diamond residues from the GC surface before the electropolymerization and spraying processes. The previously mentioned steps were the same for both SWCNT- and PANI-modified sensors.

#### 2.2.1. SWCNT Sensor

25 mg of the purified and dried SWCNTs was powdered in a marble mill and then dispersed in 10 mL of Milli-Q water containing 100 mg of sodium dodecyl sulphate (SDS) to provide the solubility of SWCNT in water. The solution was sonicated for 30 min at 2 s^−1^ in order to achieve the maximum homogeneity of the dispersion. The sonicated solution was sprayed with approximately 1 bar pressure onto the glassy carbon surface under a high temperature (approx. 200°C) air blower by spraying 35 times, dipping the surface into Milli-Q water under stirring conditions at intervals of 5 sprays so as to eliminate the SDS as its presence decreases the conductance of the SWCNT network. We deposited a layer of about 30 *μ*m thickness (measured with SEM) of purified SWCNT onto the polished tip of a glassy carbon (GC) surface. Lastly, to ensure the removal of the SDS, the SWCNT sprayed GC rods were placed into CVD furnace at 300°C for 1 h under low air flow. The carboxylic groups of the SWCNT were activated with a solution containing 200 mM EDC and 50 mM NHS [25] (dissolved in MES buffer, 50 mM and pH 5.0). The GC surface containing the SWCNTs was dipped in this solution for 30 minutes. To covalently link the TBA to the walls of the SWCNTs through the nucleophilic attack of the amine to the activated carboxylic group, the sensor subsequently was dipped overnight in a solution containing 0.001 M PBS and 1 *μ*M 5′-amine-TBA.

#### 2.2.2. PANI Sensor

Polyaniline films were electropolymerized onto the polished distal end of the glassy carbon rod via cyclic voltammetry with a three-electrode system that consisted of an Ag/AgCl reference electrode, a platinum wire as a counter electrode, and a GC rod as a working electrode. Prior to electropolymerization, GC rods were pretreated with 0.85 V potential during 10 s for the activation of the surface. Electropolymerization of aniline was carried out potentiodynamically on the GC rod within the potential range from −0.15 to 0.85 V in 0.5 M H_2_SO_4_ solution by applying 50 potential cycles at a sweep rate of 100 mV/s. Finally, the PANI-modified GC probe is dipped into 5 *μ*M TBA solution overnight for the immobilization of the thrombin aptamer via aromatic substitution at the conducting polymer surface [21], obtaining in this way the potentiometric PANI aptasensor.

Both types of sensors were washed out thoroughly with Milli-Q water to get rid of noncovalently attached aptamers.

### 2.3. Measurements

Cyclic voltammetry measurements for both electropolymerization of PANI and ligand density calculations were performed in a single compartment electrochemical cell with a 10 mL volume. Supporting electrolytes, H_2_SO_4_ and hexaammineruthenium, were deoxygenated via purging with nitrogen gas for 10 min prior to measurements, and nitrogen was bubbled during the experiments. Potentiometric measurements were conducted in 5 mL 5 mM PBS solution (pH = 7.5). This was important as to maintain the physiological pH level for the aptamers and the thrombin. The solution was stirred during the measurements at 1000 rpm. The two-electrode system consisted of an Ag/AgCl reference electrode and the developed GC sensor as the working electrode. 

#### 2.3.1. Surface Density Measurements of TBA

The number of the probe TBA-molecules that are covalently immobilized onto PANI was calculated from the number of cationic redox molecules of hexaammineruthenium forming ionic pairs with the anionic TBA backbone. Charge compensation is provided for the anionic phosphate groups in TBA by cations, typically Na^+^, K^+^, and H^+^. These cations readily exchange with other cations in the media [26] because the association constant between cations and TBA phosphate increases with the cation charge [27]. When a TBA sensor is placed in a low ionic strength electrolyte containing a multivalent cation, this latter cation exchanges with the native cation and becomes electrostatically trapped at that interface [28]. The trapped multivalent cation, hexaammineruthenium in our case, due to its oxidizing character can be readily reduced at the electrode as a surface-confined species. The resulting charge at the surface can easily be calculated from the cyclic voltammogram by integrating the suitable reduction peak. In saturation conditions, the amount of surface-linked hexaammineruthenium is proportional to the surface density of aptamers attached to the carbon nanotubes and PANI substrates. The objective was to characterize the electrode surface by determining the total number and the density of the TBA in both sensor surfaces and to explain the sensitivity and stability differences that are observed between the two studied sensors.

#### 2.3.2. Potentiometric Setup

The EMF values recorded with TBA-modified SWCNTs and PANI sensors were measured against the Ag/AgCl reference electrode with a Lawson multi-channel potentiometer in 5 mM PBS solution to maintain the low ionic strength. For the thrombin detection assay, the potentiometric cells were introduced in a solution in which successive aliquots of thrombin solutions were added giving rise to a total thrombin concentration starting from 0.5 nM up to 800 nM, which is approximately the maximum physiological levels in blood [29, 30]. Assays were performed at solution temperatures of 37°C. 

## 3. Results and Discussion

### 3.1. Surface Ligand Density in Functionalized PANI and SWCNTs

**Figure 1:**
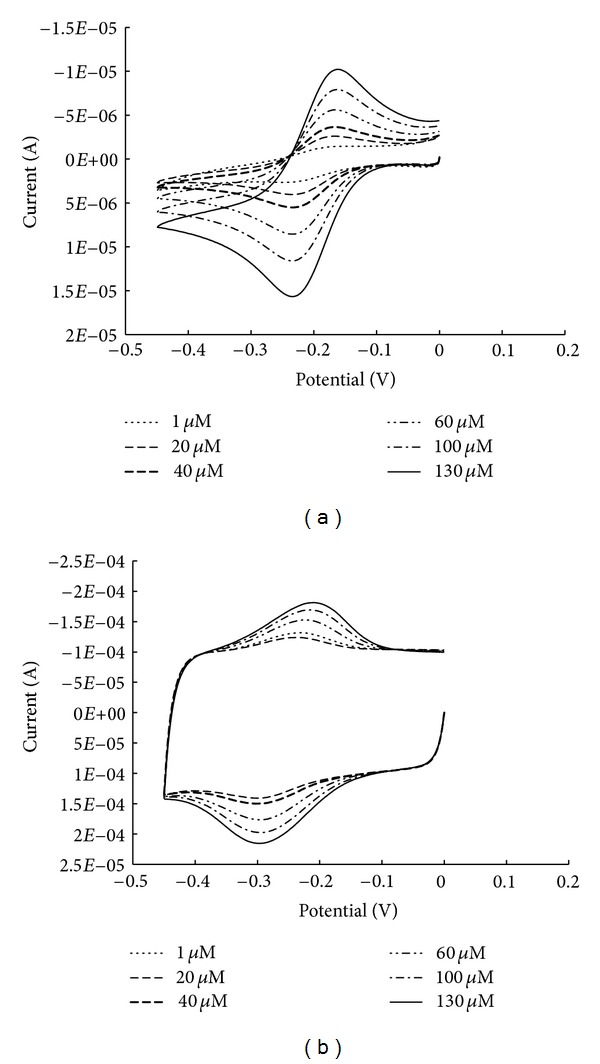
CVs of the (a) PANI- and (b) SWCNT-based aptasensors at different hexaammineruthenium concentrations. In all cases, the sweep rate was 0.1 V/s. The observed area under the average cathodic peak from 1 *μ*M to 130 *μ*M due to addition of hexaammineruthenium is used for calculations in (1) and (2).

Voltammetric Γ_RU_ is a direct measure of the charge density (in mol/cm^2^) of [Ru(NH_3_)_6_]^3+^ forming ion pairs with the phosphate groups of the TBA-modified sensors [24, 31, 32]. It can be calculated from

(1) $$\begin{array}{c} \Gamma_{\text{Ru}}=\frac{Q}{nFA}, \end{array}$$

where *Q* is the charge obtained by the integration of the redox peaks in the cyclic voltammograms of the hexaammineruthenium coordination complex at saturation point (see Figure 1), *n* is the number of electrons in the reduction reaction from Ru(III) to Ru(II), *F* is the Faraday constant, and *A* is the area of the working electrode.

The calculated Γ_Ru_ value can be directly converted to the TBA surface density, Γ_TBA_, in molecule/cm^2^ using the relationship

(2) $$\begin{array}{c} \Gamma_{\text{TBA}}=\Gamma_{\text{RU}}\left(\frac{z}{m}\right)N_{A}, \end{array}$$

where *m* is the number of nucleotides in the TBA, *z* is the charge of the hexaammineruthenium redox species, which is 3 in our case, and *N*_*A*_ is Avogadro's number. In order to determine the surface ligand density, 1 mM stock hexaammineruthenium solution is added to the voltammetric cell starting from 1 *μ*M up to 130 *μ*M hexaammineruthenium in the presence of 5 mM PBS until it is saturated as shown in Figure 1. For each successive step, one CV cycle is recorded after the addition of the corresponding amount of hexaammineruthenium solution without any further modification to the electrochemical cell. All of the CVs are overlaid and shown for PANI (Figure 1(a)) and SWCNT (Figure 1(b)). 

According to the data in Figure 1, in both sensors, the CV has cathodic (reduction) and anodic (oxidation) peaks associated with hexaammineruthenium, but the reduction peak shifted negatively compared to hexaammineruthenium with TBA, supporting that the signal originates from hexaammineruthenium bound in the TBA [33]. Ideally, there should be no cathodic and anodic peak separations for a surface-confined molecule. Peak separation can be induced by kinetic control or interfacial electron-transfer rates comparable to the scan rate. Other reasons for apparent “nonideality” could be dissociation and association of [Ru(NH_3_)_6_]^3+/2+^ that accompanies the electrochemical process due to different stoichiometric PO_4_^3−^/hexaammineruthenium ratios when hexaammineruthenium is in the oxidized and the reduced states [34]. These results also indicate that the hexaammineruthenium is slightly more present at PANI surface. The typical capacitive behavior of the carbon nanotube sensor is observed in Figure 1(b). The area under the cathodic peak (subtracting the capacitive contribution) is related to the total amount of surface-confined hexaammineruthenium complex that is reduced. According to the areas obtained under the PANI and the SWCNTs peaks, there is a much higher charge accumulation on SWCNT. These results could be due to the larger surface area of the carbon nanotubes and to the higher surface area density of the thrombin aptamer covalently linked to the latter surface. 

Average of the saturation charge value at 130 *μ*M is used to calculate the hexaammineruthenium charge density, Γ_RU_ (mol/cm^2^). This value is 5.18 × 10^−12^ mol/cm^2^ and 5.19 × 10^−12^ mol/cm^2^ for PANI and SWCNTs sensors, respectively. Placing this value in (2) gives the total surface aptamer density per cm^2^. After estimating the approximate area of each sensor (see (3)), the aptamer density on the surface can be easily calculated.

According to IUPAC [35, 36], surface area of a nonmetallic porous electrode surface can be estimated by determining the apparent total capacitance of the electrode surface and assuming that the double layer charging, that is, the capacitive component, is the only process in the conditions where voltammetric curves are recorded. We have estimated the total charge (*Q*) by the integration of the average cathodic peaks starting from 1 *μ*M to 130 *μ*M in the cyclic voltammograms of the hexaammineruthenium in Figure 1 and subsequently calculated the total capacitance, *C*_T_, by dividing it by the sweep rate following the expression *C*_T_ = *δQ*/*δE* = *Iδt*/*δE* = *I*/(*δE*/*δt*). The area of the sensor is obtained by dividing the estimated total capacitance, *C*_T_, by the empirical reference value, C* (10 *μ*F/cm^2^), used for the capacitance of the purified SWCNTs [37], as follows:

(3) $$\begin{array}{c} A=\frac{C_{\text{T}}}{C^{*}}. \end{array}$$

Table 1 shows the values obtained from the addition of hexaammineruthenium to PANI- and SWCNTs-based aptasensors. The obtained results show considerable differences in the calculated surface areas for both sensors: 164.29 cm^2^ for SWCNTs versus 11.27 cm^2^ for PANI with standard deviations of 25.7 cm^2^ and 2.16 cm^2^ for SWCNTs and PANI, respectively (*N* = 3). The area of the polished glassy carbon surface is 0.07 cm^2^. The increase in the area of PANI sensor is thought to be due to the polymer chains conformation on the surface which is creating some slight roughness compared to bare GC surface. The area differences between PANI and SWCNTs sensors could result from the fact that the surface area to volume ratio of SWCNTs is much higher than that of the PANI chains deposited in the two-dimensional plane of the sensor surface. Furthermore, spaghetti-like formation [16] of the SWCNTs compared to very orderly distributed PANI chains also supports this result. The total charges on the surface are calculated as 5.63 × 10^−6^ C for PANI- and 8.21 × 10^−5^ C for SWCNT-based sensors with standard deviations of 1.08 × 10^−06^ C and 1.29 × 10^−05^ C for PANI and SWCNTs, respectively (*N* = 3). Using (1) and (2), 6.23 × 10^+11^ and 6.24 × 10^+11^ TBA molecules are bound per cm^2^ of PANI and SWCNT surfaces, respectively, which lead to a total of 7.03 × 10^+12^ aptamer molecules on PANI and 1.03 × 10^+14^ aptamer molecules on SWCNT total sensor surfaces. All of the corresponding standard deviations are presented in Table 1.

### 3.2. Potentiometry

Potentiometric responses of PANI- and SWCNTs-based TBA-modified sensors against thrombin are evaluated. Evaluation is done under consideration of that the sensing system is not based on equilibrium process; hence, it should not be explained by the Nernstian theory. The sensors do not contain any membrane to provide the equilibrium process that is necessary for realizing the Nernstian theory, and they are similar to the field effect transistors (FETs), which are also considered potentiometric sensors [4, 5]. Instead, our target is a neutral protein, where the isoelectric point is in the pH range of 7,0–7,6. What is thought is that the conformational change of the aptamer during binding event changes the capacitance value of the surroundings of the SWCNTs/PANI (the transducing elements) which leads to the detectable signal. Within this scenario, the response could be considered sensitive enough, at least for any type of semiquantitative or qualitative detection, since the target analyte is a protein that shows an illness above or below a critical level in blood. 

**Figure 2:**
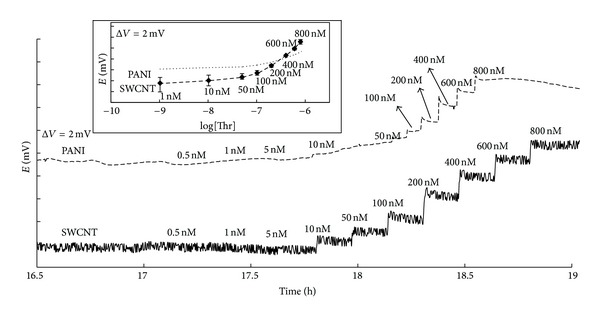
Potentiometric response of the TBA-modified PANI- and SWCNT-based sensors to thrombin. Concentration range is 0,5 nM–800 nM in both cases. The inset shows average calibration curves for the PANI and the SWCNTs potentiometric sensors against thrombin with the corresponding error bars.

The stabilization time for the PANI sensor is much longer (16 hours) than the approximately 30 minutes needed for the carbon nanotube sensor using the same experimental conditions. It might be related to the time needed to reach the equilibrium position of the nucleic acid segment onto the different surfaces and to the establishment of the interfacial double layer in PANI sensor rather than the SWCNT sensor. This could also be attributed to the relatively lower chemical stability and higher light sensitivity of PANI against SWCNTs.  Figure 2 shows the potentiometric responses obtained when increasing the total concentration of thrombin in the solution. Both sensors are kept without analyte until reaching a stable state, and additions have been made simultaneously in both sensors for technical reasons. The sensitivity is calculated as 2.97 mV/decade and 8.03 mV/decade for the PANI and SWCNTs aptasensors, respectively. These results are in accordance with the higher surface density of the aptamers in the SWCNT potentiometric sensor. The inset of Figure 2 shows average (*N* = 3) calibration curves of the potentiometric response for both SWCNT and PANI. 

Selectivities of both sensors were measured against elastase and BSA separately. Both sensors did not show a noticeable response until 2 *μ*M level. Additions were done first by ranging from 0.5 *μ*M up to 800 nM as in the sensitivity experiments with no noticeable response. Later, the additions have been conducted by first 1 *μ*M and later 2 *μ*M of interfering proteins, where they showed a noticeable signal. 

Comparing the performances of PANI and SWCNTs as transducers in potentiometric aptasensors, the first aspect is that PANI can be deposited electrochemically onto the GC surface with a very high control on thickness. The same thickness control is difficult to reach with carbon nanotubes using any of the available deposition techniques [38]. Nevertheless, the produced sensor interface is very different in both sensors: while we obtain a quite homogeneous surface with PANI (area = 11.27 cm^2^), the surface of the spaghetti-like deposited carbon nanotubes is very rough and inhomogeneous producing a very large superficial interface (area = 164.29 cm^2^). 

**Scheme 1:**
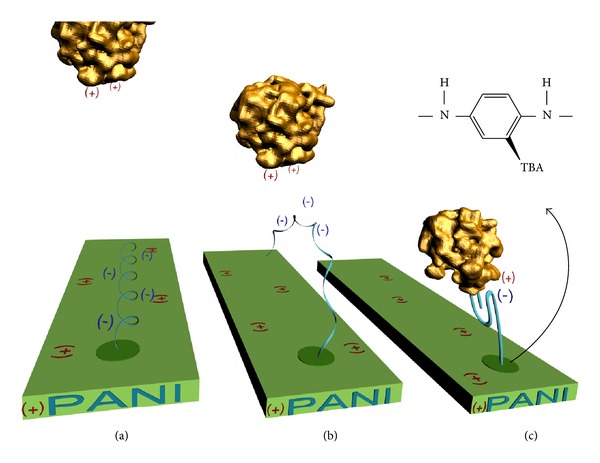
Scheme depicting charge competition and TBA-thrombin binding. (a) When the thrombin is not in the system, TBA tends to remain attached to PANI backbone due to charge attraction. (b) TBA starts to dislocate from PANI surface through thrombin. (c) Positively charged active site of the thrombin binds to the TBA which leads to a potentiometric signal.

The chemistry used to covalently immobilize the thrombin aptamer onto the substrate gives very similar results in terms of surface density of the ligand (Γ_TBA_ is approximately 6.2 × 10^+11^ molecule/cm^2^ in both sensors). While the aromatic substitution involving a thiol group is used to link aptamers to PANI (see Scheme 1), the covalent bonds via carboxylic groups have been established between the receptors and SWCNTs. However, the very different available surface area in both substrates gives rise to large differences in the total ligand linked to the substrate (*N*_TBA_ = 7.03 × 10^+12^ in PANI and 1.03 × 10^+14^ in SWCNTs). 

Considering that SWCNTs have a larger total surface area than the relatively planar PANI surface, a higher percentage of the immobilized and active aptamers is probably responsible for the differences in the observed sensitivity. Another reason for the higher sensitivity could be attributed to the relatively higher affinity that is caused by the covalent backbone of the carboxylic acid-amine interaction. Also, there are some electrostatic interactions present which caused the phosphate backbone of the TBA and positively charged surface of PANI. These interactions must be higher than the ones between TBA and CNTs so that the total energy needed for the TBA to get its chair G-quartet formation is lower in the case of CNTs which are thought to be the source of the potential response [39]. Further research is needed to be able to explain this difference considering the similarities and differences in both charge transfer mechanisms. However, both sensors have a similar limit of detection (LOD) (80 nM for SWCNT-based sensor and 71 nM for PANI-based sensor) values. Relatively lower noise level in PANI-based sensor is thought to be responsible for this slightly better LOD.

To conclude, using a redox molecule to determine the total charge on the surface of TBA-modified sensor and as a consequence being able to calculate the surface ligand density can help in enhancing sensor's performance. However, the current method does not provide information on the formation and the distribution of the aptamer molecules on the SWCNT/PANI surface. Further investigation is needed to determine the correct formation of the aptamers to thoroughly understand the underlying phenomena of the generated EMF. In comparison to the previously reported CNT-based potentiometric sensor [16], PANI-based sensor does provide neither good sensitivity nor stability. However, both sensors responded to interfering proteins exhibiting a similar selective behavior.

## Figures and Tables

**Table 1:** Calculated surface charge and ligand density values with the corresponding standard deviations (*N* = 3).

	*A* (cm^2^)	*Q* (C)	Γ_RU_ (mol/cm^2^)	Γ_TBA_ (molecule/cm^2^)	*N*_TBA_ at sensor surface
PANI	11.27	5.63 × 10^−06^	5.18 × 10^−12^	6.23 × 10^+11^	7.03 × 10^+12^
SWCNT	164.29	8.21 × 10^−05^	5.19 × 10^−12^	6.24 × 10^+11^	1.03 × 10^+14^
PANI St. dev.	2.16	1.08 × 10^−06^	9.94 × 10^−13^	1.20 × 10^+11^	1.00 × 10^+12^
SWCNT St. dev.	25.7	1.29 × 10^−06^	8.11 × 10^−13^	9.77 × 10^+10^	1.60 × 10^+13^
